# Genomic characterization of multidrug-resistant *Escherichia coli* with PBP3 insertions carrying *bla*_NDM-5_ carbapenemase isolated from infants in Kenya

**DOI:** 10.1128/mra.00121-25

**Published:** 2025-06-18

**Authors:** Kevin Kariuki, Collins Kigen, James Wachira, Polycarp Mogeni, Doreen Rwigi, Benson Singa, Kirkby D. Tickell, Ferric C. Fang, Samuel Kariuki, Judd L. Walson, Patricia B. Pavlinac

**Affiliations:** 1 Centre for Microbiology Research, Kenya Medical Research Institute 118982, Nairobi, Kenya; 2 Centre for Clinical Research, Kenya Medical Research Institute 118982, Nairobi, Kenya; 3 Department of Emerging Infectious Diseases, USAMRD—Africa, Nairobi, Kenya; 4 Department of Global Health, University of Washington 7284, Seattle, Washington, USA; 5 The Childhood Acute Illness & Nutrition (CHAIN) Network, Nairobi, Kenya; 6 Department of Medicine, Division of Allergy and Infectious Diseases, University of Washington 7284, Seattle, Washington, USA; 7 Department of Laboratory Medicine and Pathology, University of Washington 7284, Seattle, Washington, USA; 8 Department of International Health, John Hopkins University, Baltimore, Maryland, USA; Portland State University, Portland, Orlando, USA

**Keywords:** metallo-beta-lactamase, *Escherichia coli*, infants, carbapenems, Kenya

## Abstract

We report on carbapenem-resistant *Escherichia coli* ST46 and 167 strains isolated from fecal samples of 4- and 8-month-old infants, respectively, in Kenya, collected at discharge after more than 4 days of hospitalization. The isolates carried *bla*_NDM-5_ carbapenemase and a PBP3 (_333_YRIN/K_334_) insertion. This finding has implications for patient management.

## ANNOUNCEMENT

Enterobacterales carrying metallo-β-lactamases (MBL) are a global public health concern due to their resistance to multiple β-lactam antibiotics that is not reversed by the β-lactamase inhibitor avibactam (1, 2). *E. coli* carrying NDM-type MBLs that also contain penicillin-binding protein 3 (PBP3) insertions have reduced susceptibility to aztreonam–avibactam and cefiderocol, considered lastline antibiotics for MBL-producing isolates (3–5). To date, 10 *E. coli* strains carrying *bla_N_*_DM-5_ have been reported in Kilifi, Nairobi, and Kisii counties in Kenya (6–8), none with PBP3 insertions.

Two *E. coli* isolates, EC3009 and EC2031, were recovered from rectal swabs of 4- and 8-month-old infants collected at discharge from a Kenyan hospital in Kisii County (0.6773° S, 34.7796° E) in March 2018 and October 2019, respectively, in a clinical trial assessing the efficacy of a 5-day course of azithromycin on post-discharge mortality (9). The rectal swabs were streaked directly onto MacConkey agar (Oxoid, United Kingdom) and incubated at 37°C for 24 h. *E. coli* isolates were identified biochemically using an API 20E strip (bioMérieux, USA) (10). Antimicrobial susceptibility testing was performed by disc diffusion, interpreted according to Clinical and Laboratory Standards Institute 2024 guidelines (11).

Carbapenem-nonsusceptible isolates were subcultured and purified on Mueller-Hinton agar (37°C, 24 h) (Oxoid, United Kingdom). Genomic DNA was extracted using the Quick DNA Fungal/Bacterial Miniprep Kit (Zymo Research, Germany) following the manufacturer’s instructions. DNA purity and concentration were assessed using Nanodrop ND-2000 (Thermo Fisher, USA) and Qubit 4 with Qubit 1× dsDNA High Sensitivity assay kit (Thermo Fisher, USA). Libraries were constructed using the Illumina DNA prep kit (Illumina Inc., USA) for (2 × 150 bp reads) and sequenced in a P1 flow cell on NextSeq 1000 (Illumina Inc., USA). Quality of the raw reads was assessed using FastQC v0.12.1 (12) and trimmed with fastp v0.20.1 (13). Reads were assembled with Shovill v1.1.0 (https://github.com/tseemann/shovill). Quality of assemblies was assessed using QUAST v5.0.2 (14) and CheckM v.1.2.3 for completeness and contamination. Strain typing was done using MLST v2.23.0 (https://github.com/tseemann/mlst). Serotype identification was done using EcOH (15) on ABRicate v1.0.1 (https://github.com/tseemann/abricate) and phylogrouping using ezclermont v0.7.0 (16). Acquired antimicrobial resistance genes (ARGs) and mutations that confer resistance were identified using AMRFinderPlus v3.12.8 (17), CARD v1.0.1, and Resfinder v.4.6.0 (18, 19). PBP3 (*ftsl*) amino acid sequence was compared to *E. coli* K-12 substr. MG1655 (U00096.3). Plasmid replicons were identified with https://github.com/tseemann/abricate, using the Plasmidfinder (20). PGAP v6.9 was used to annotate the genome sequences (21) and visualized using SnapGene v8.0.2. All tools were used in default settings.

**Fig 1:**
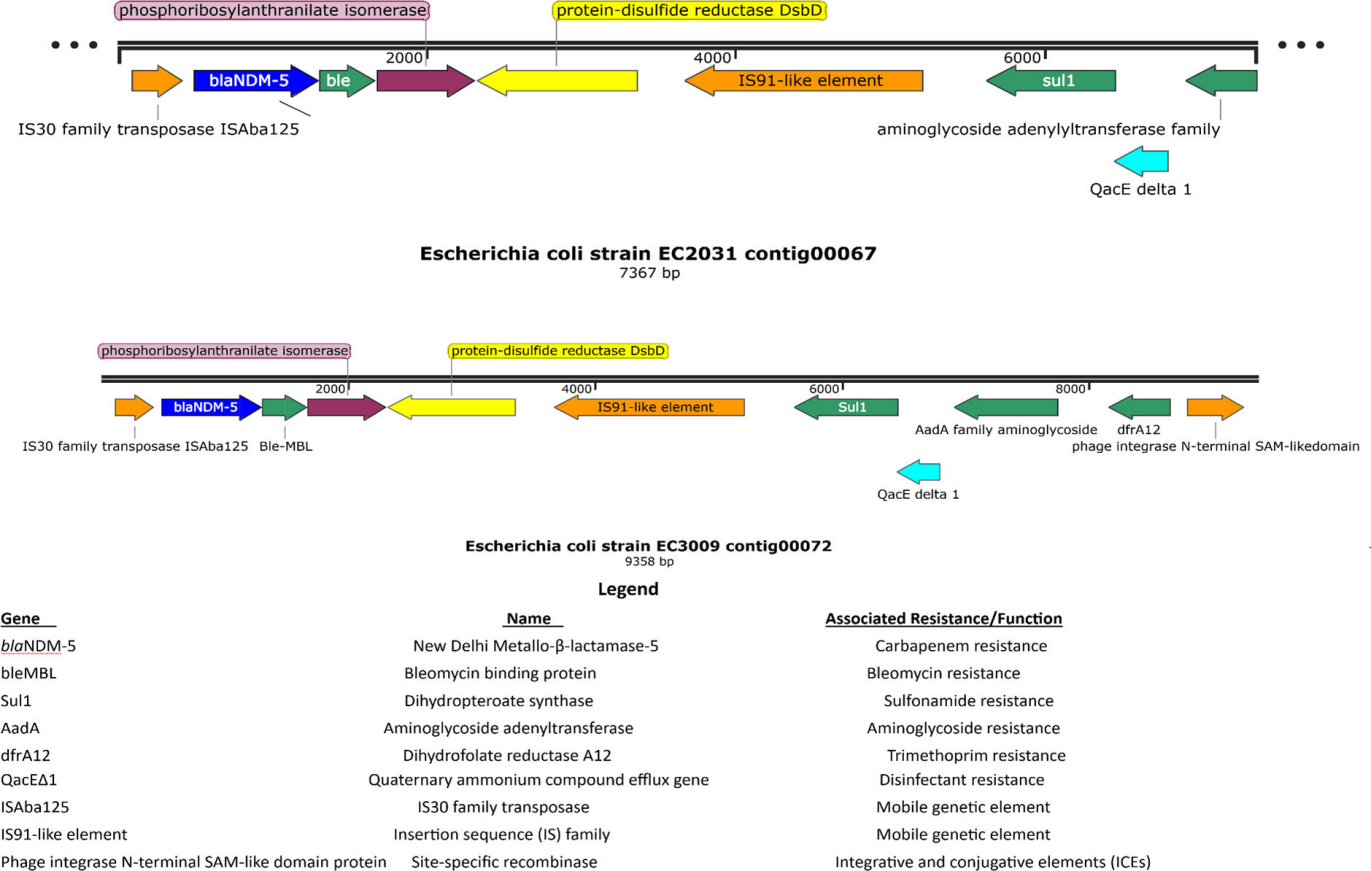
Genome organization of *E. coli* strains EC2031 and E3009. Linear genome context of *bla*_NDM-5_ (highlighted in blue). Arrows indicate open reading frames, with arrowheads indicating the direction of transcription: green, antibiotic-resistance-encoding genes; blue, New Delhi Metallo-β-lactamase-5 (*bla*_NDM-5_), orange, transposon- and integron-associated genes; electric blue, antiseptic-resistance-encoding genes, yellow, DsbD, genes encoding redox reactions; purple, tryptophan biosynthesis genes.

Phenotypic, genome features, sequence types (ST), and phylogroups are provided in Table 1. The strains belonged to ST46 and ST167, predominantly associated with *bla*_NDM_ both globally and in Kenya (6). ARGs and mutations associated with resistance to multiple antibiotic classes were detected in both isolates (Table 1). Plasmid replicon types identified in EC3009 include IncFII, IncFIA, IncFIB, associated with *bla*_NDM-5_, and Col (BS512), while EC2031 had IncFIA and Col (BS512). Linear comparison showed high genetic context similarity with *bla*_NDM-5_ flanked by IS30 and IS91 with *ble*MBL encoding bleomycin resistance (Fig. 1).

**TABLE 1:** Phenotypic and genome characteristics of two *E. coli* carrying *bla_NDM-5_* strains isolated in Kenya

Strain	EC2031	EC3009
Phenotypic antibiotic resistance profile	Chloramphenicol (R), gentamicin (R), cefoxitin (R), cefotaxime (R), ampicillin (R), azithromycin (R), ceftazidime (R), ciprofloxacin (R), and imipenem (I)	Chloramphenicol (R), gentamicin (R), cefoxitin (R), cefotaxime (R), ampicillin (R), azithromycin (R), ceftazidime (R), ciprofloxacin (R), and imipenem (R)
Total raw reads	4,720,342	4,568,674
Genome size (bp)	4,926,003	4,710,358
N50 value (bp)	137193	94376
No. Contigs	117	127
Coverage (x)	140.6	143.1
GC content (%)	50.80%	50.70%
Genome completeness	99.96	99.96
Genes (total)	4,972	4,764
CDSs (total)	4,849	4,646
Genes (coding)	4,594	4,351
CDSs (with protein)	4,594	4,351
Genes (RNA)	123	118
Multilocus sequence type (ST)	167	46
Phylogroup	A	A
Serotype	H5	O9:H10
Antibiotic resistance genes	Beta-lactams (*bla*_OXA-1_, *bla*_CTX-M-15_, *bla*_TEM-1B_, PBP3 _333_YRIN/K_334_ insertion), fluoroquinolones (*gyrA_S83L*, *gyrA_D87N*, *parC_S80I*), macrolides (*ermB*, *mphA*), aminoglycosides (*aac (3)-IIa*, *rmtB*), sulfonamides (*sul1*), and tetracycline (*tetA*)	Beta-lactams (*bla*_OXA-1_, *bla*_CTX-M-15_, *bla*_TEM-1B_, PBP3 _333_YRIN/K_334_ insertion), fluoroquinolones (*gyrA_S83L*, *gyrA_D87N*, *parC_S80I*), macrolides (*ermB*, *mphA*), aminoglycosides (*aac (3)-IIa*, *rmtB*), sulfonamides (*sul1*), and tetracycline (*tetA*)
Accession number	JBJLZZ010000000	JBJMAA000000000

## Data Availability

This whole-genome shotgun project has been deposited at GenBank under accession number PRJNA1192017. Raw sequence reads are available under SRA accession numbers SRR31548417 and SRR31548418.
